# Renal denervation in 2025: long-term evidence supporting sympathetic modulation as a durable strategy for uncontrolled hypertension

**DOI:** 10.1016/j.ijcrp.2026.200587

**Published:** 2026-02-02

**Authors:** Dominique Stephan, Emma Morisot, François Bronner, Eric Prinz, Mihaela Calcaianu, Elena-Mihaela Cordeanu

**Affiliations:** a Service d'HTA et Maladies Vasculaires, Hôpitaux Universitaires de Strasbourg, France; b Service de Cardiologie, Hôpitaux Universitaires de Strasbourg, France; c Service de Néphrologie, Hôpitaux Universitaires de Strasbourg, France; d Service de Cardiologie, GHRMSA, Mulhouse, France

**Keywords:** Renal denervation, Uncontrolled hypertension, Sympathetic nervous system

## Abstract

**Background:**

Renal denervation (RDN) has emerged as a promising device-based therapy for uncontrolled hypertension. Recent data from major cardiovascular conferences (EuroPCR, ESH, TCT 2025) provide unprecedented 3-year follow-up evidence on efficacy, safety, and clinical outcomes.

**Methods:**

We synthesize key findings from the SPYRAL HTN-ON MED 3-year final report, the Global Symplicity Registry DEFINE (GSR-DEFINE) real-world data, SPYRAL AFFIRM post-approval study, and pooled analyses on acute hypertensive events. Data sources are distiguisehed by level of evidence: randomized sham-controlled trials versus observational registry data.

**Results:**

At 3 years, SPYRAL HTN-ON MED demonstrated sustained office systolic blood pressure (SBP) reductions of −18.5 mmHg (RDN) versus −11.7 mmHg (sham), with treatment difference of −6.8 mmHg (p = 0.0002). The GSR-DEFINE registry (n = 3109) showed −20.5 mmHg office SBP reduction at 3 years with progressively increasing effect over time. Pooled analysis revealed a 43% reduction in acute hypertensive events with RDN. No renal artery stenosis or need for reintervention was observed through 3 years.

**Conclusion:**

These data support RDN as a potentially durable and safe adjunctive therapy for uncontrolled hypertension in appropriately selected patients. Benefits appear to extend to high-risk populations including patients with diabetes, chronic kidney disease, and prior stroke, though long-term cardiovascular outcome data remain awaited.

## 1 Introduction

Hypertension remains the leading modifiable risk factor for cardiovascular disease worldwide, yet blood pressure (BP) control rates remain suboptimal despite major pharmacological advances [1]. Contemporary data indicate that nearly half of patients with hypertension fail to achieve recommended BP targets, owing to a combination of poor medication adherence, drug intolerance, therapeutic inertia, and true treatment resistance. This persistent gap between guideline-recommended targets and real-world BP control underscores the need for adjunctive non-pharmacological strategies. Catheter-based renal denervation (RDN), which attenuates sympathetic nervous system activity through radiofrequency ablation of the renal nerves, has emerged as a promising device-based therapeutic option. By targeting a key pathophysiological mechanism of hypertension, RDN offers sustained BP reduction independent of medication adherence. For clarity, "uncontrolled hypertension" refers to blood pressure above target despite treatment, while "resistant hypertension" specifically denotes uncontrolled BP despite three or more antihypertensive agents at optimal doses, including a diuretic, after exclusion of secondary causes and pseudoresistance. The studies reviewed herein include both populations, as specified in their respective inclusion criteria. The 2023 European Society of Hypertension (ESH) guidelines introduced renal denervation as a complementary treatment strategy for selected patients with uncontrolled hypertension at high cardiovascular risk, particularly those with true resistant hypertension despite treatment with three or more antihypertensive agents, including a diuretic [2]. These recommendations have subsequently been reinforced by international expert consensus statements and position papers. Importantly, the 2024 European Society of Cardiology (ESC) Guidelines on the management of arterial hypertension further recognize renal denervation as a validated adjunctive therapy in carefully selected patients with uncontrolled or resistant hypertension, emphasizing its role within a structured, multidisciplinary hypertension care pathway [3]. Both ESH 2023 and ESC 2024 guidelines emphasize that RDN should be performed within a framework of shared decision-making, with patients fully informed about the procedure, expected benefits, and current evidence limitations. Furthermore, both guidelines recommend that RDN be performed in specialized, experienced centers with established hypertension programs to ensure appropriate patient selection and procedural quality. The accumulation of robust sham-controlled trials and emerging long-term outcome data, presented at major cardiovascular congresses in 2024–2025, has consolidated RDN as a credible addition to the contemporary therapeutic armamentarium against hypertension. It should be noted that some data presented at these conferences are currently available only in abstract form and have not yet undergone formal peer-reviewed publication. This reflects the nature of a "Highlights from the Literature" article, which aims to provide timely synthesis of emerging evidence.

## 2 Spyral HTN-ON MED: three-year final report

The SPYRAL HTN-ON MED trial is a global, randomized, sham-controlled study evaluating radiofrequency RDN using the Symplicity Spyral catheter in hypertensive patients prescribed up to three antihypertensive medications [4]. Medication adherence was rigorously verified through witnessed drug intake and urine/blood toxicology screening, providing robust confirmation of true uncontrolled hypertension. The 3-year final report, presented at TCT 2025, represents the longest follow-up of any sham-controlled RDN trial to date. At 36 months, patients randomized to RDN demonstrated significantly greater BP reductions compared to sham (Table 1). Office systolic BP decreased by −18.5 mmHg in the RDN group versus −11.7 mmHg in the sham group, yielding a treatment difference of −6.8 mmHg (p = 0.0002). Twenty-four-hour ambulatory BP monitoring (ABPM) confirmed these findings, with reductions of −14.0 mmHg (RDN) versus −9.3 mmHg (sham), representing a treatment difference of −4.7 mmHg (p = 0.0028). Notably, BP control (<140 mmHg) was achieved in 47% of RDN patients compared to only 22% in the sham group, representing a 2.1-fold improvement. The safety profile was excellent, with no significant difference in the composite safety endpoint between groups (3.3% RDN vs 2.7% sham, p > 0.99). Critically, no renal artery stenosis >70% was observed in any patient, and no renal reinterventions were required through 3 years of follow-up. The magnitude of the treatment effect was sustained without evidence of attenuation, and 74% of sham patients elected to cross over to RDN at 6 months, indicating patient willingness to undergo the procedure after unblinding,

**Table 1:** SPYRAL HTN-ON MED 3-Year efficacy and safety results.

Outcome	RDN (n = 184)	Sham (n = 113)	P-value
Office SBP change (mmHg)	−18.5	−11.7	0.0002
24-h ABPM SBP change (mmHg)	−14.0	−9.3	0.0028
BP control <140 mmHg (%)	47%	22%	–
Composite safety endpoint (%)	3.3%	2.7%	>0.99
Renal artery stenosis >70%	0%	0%	–

ABPM: ambulatory blood pressure monitoring; BP: blood pressure; SBP: systolic blood pressure. Data from Kandzari et al., TCT 2025.

**Table 2:** GSR-DEFINE 3-Year outcomes and temporal evolution of blood pressure reduction.

Timepoint	6 months	1 year	2 years	3 years
n	2.847	2.156	1.423	892
Office SBP (mmHg)	−16.5	−17.5	−18.9	−20.5
24-h ABPM (mmHg)	–	–	–	−9.7
BP control <140 (%)	–	–	–	37%

Baseline: n = 3109; mean office SBP 170 mmHg; 4.7 medications; 41% diabetes. Sample sizes at each timepoint reflect patients with available follow-up data. Data from Mahfoud et al., EuroPCR 2025.

81 though this may reflect multiple factors including symptom burden and expectations.

## 3 GSR-Define: Real-world evidence at three years

The Global Symplicity Registry DEFINE (GSR-DEFINE) is a prospective, all-comer observational study conducted across 251 sites in 55 countries, representing the largest real-world RDN dataset worldwide [5]. As an observational registry, these data carry inherent limitations including selection bias, absence of a control group, potential regression to the mean, and reliance on physician-reported medication without objective adherence verification. These limitations should be considered when interpreting the magnitude of BP reductions. At EuroPCR 2025, investigators reported 3-year outcomes in 3109 patients meeting ESC eligibility criteria for RDN (uncontrolled hypertension at high cardiovascular risk). Baseline characteristics reflected a high-risk population: mean office SBP 170 mmHg, 4.7 antihypertensive medications, and 41% prevalence of diabetes mellitus. At 3 years, office SBP decreased by −20.5 mmHg (p < 0.0001) and 24-h ABPM by −9.7 mmHg (p < 0.0001).BP control (<140 mmHg) improved from 1.5% at baseline to 37% at 3 years. The effect was progressive over time, with office SBP reductions of −16.5 mmHg at 6 months, −17.5 mmHg at 1 year, −18.9 mmHg at 2 years, and −20.5 mmHg at 3 years, suggesting no attenuation of the treatment effect. However, this progressive increase should be interpreted cautiously, as alternative explanations beyond true biological amplification include improved medication adherence over time, survivor bias (loss of non-responders to follow-up), and diminishing white-coat effect. Antihypertensive medication burden remained stable (4.65–4.52 medications, p = 0.0001), indicating that BP reductions were not explained by medication intensification (see Table 2).

## 4 Efficacy in high-risk populations

A subgroup analysis of patients with prior stroke (n = 301) from GSR-DEFINE, presented at ESH 2025, demonstrated that RDN is effective in this very high-risk population (57% cardiac disease, 46% diabetes, 30% chronic kidney disease). Office SBP decreased by −21.4 mmHg and 24-h ABPM by −11.6 mmHg at 3 years (both p < 0.0001), with a similar temporal pattern of progressive BP reduction (Table 3).

**Table 3:** Blood pressure reductions in high-risk subgroups (SPYRAL AFFIRM, 6 months).

Subgroup	Office SBP (mmHg)	Home SBP (mmHg)
Type 2 diabetes (n = 118)	−13.3	−5.9
CKD stage 3b (n = 24)	−19.3	−5.4
Isolated systolic HTN (n = 140)	−10.6	−4.6

CKD: chronic kidney disease; HTN: hypertension; SBP: systolic blood pressure. Data from Mahfoud et al., TCT 2025.

The SPYRAL AFFIRM post-approval study, presented at TCT 2025, evaluated RDN in 210 patients with high cardiovascular risk at 6 months. Patients with type 2 diabetes (n = 118) achieved office SBP reduction of −13.3 mmHg and home BP reduction of −5.9 mmHg. Patients with stage 3b chronic kidney disease (eGFR 30–45 mL/min/1.73 m^2^, n = 24) demonstrated substantial BP reductions (−19.3 mmHg office, −5.4 mmHg home) with stable renal function. Patients with isolated systolic hypertension (diastolic BP < 90 mmHg, n = 140) also responded favorably (−10.6 mmHg office, −4.6 mmHg home).

## 5 Clinical outcomes: reduction in acute hypertensive events

A pooled analysis of SPYRAL HTN-OFF MED and SPYRAL HTN-ON MED (n = 703), presented at ESH 2025, evaluated the incidence of acute hypertensive events defined as SBP ≥180 mmHg or DBP ≥120 mmHg, hypertensive crisis, or hypertensive symptoms with SBP >150 mmHg. This composite endpoint encompasses both hypertensive emergencies (with acute target organ damage) and hypertensive urgencies (without acute target organ damage). At 6 months, the incidence was significantly lower in the RDN group (2.9%) compared to sham (7.9%), representing a 43% relative risk reduction (HR 0.57, 95% CI 0.38–0.86, p = 0.0077). This finding provides direct evidence that BP reductions achieved with RDN translate into clinically meaningful reductions in acute hypertensive events.

## 6 Discussion

The 2025 data consolidate RDN as a promisingtherapeutic option for uncontrolled hypertension. Several key observations emerge from this evidence synthesis. First, the BP-lowering effect of RDN appears durable, with consistent and even progressive reductions maintained through 3 years without need for reintervention. Second, the safety profile is reassuring, with no signal of renal artery stenosis or deterioration of renal function. Third, RDN appears effective across diverse high-risk populations, including patients with diabetes, chronic kidney disease, prior stroke, and isolated systolic hypertension. Fourth, BP reductions translate into meaningful clinical benefits, as evidenced by the reduction in acute hypertensive events. However, important limitations and uncertainties warrant discussion. First, while the 43% reduction in acute hypertensive events is clinically meaningful, current trials were not powered to detect differences in hard cardiovascular endpoints such as stroke, myocardial infarction, or mortality. The observed reduction in acute events represents a surrogate marker suggesting potential downstream benefit, but definitive cardiovascular outcome trials are needed before BP reductions can be assumed to translate into reduced stroke risk or mortality. Second, despite overall positive results, a substantial proportion of patients do not achieve BP control after RDN. In SPYRAL HTN-ON MED, 53% of RDN patients did not achieve office BP < 140 mmHg at 3 years, and in GSR-DEFINE, 63% remained uncontrolled. These figures underscore that RDN is not universally effective and that patient selection remains a challenge. Currently, there are no reliable pre-procedural predictors of response. Research into biomarkers of sympathetic overactivity and imaging of renal nerve distribution aims to improve responder identification. Clinicians should counsel patients that while many experience meaningful BP reduction, a significant minority may not respond adequately. Third, inter-individual variability in BP response remains poorly understood, and challenges in identifying likely responders prior to the procedure persist. These findings support the integration of RDN into comprehensive hypertension management pathways, particularly for patients with uncontrolled BP despite optimized pharmacotherapy. The procedural simplicity, favorable safety profile, and durability of effect position RDN as a potentially valuable tool for cardiovascular risk reduction in the growing population of patients with inadequately controlled hypertension.

**Limitations and perspectives.** This Highlight primarily synthesizes long-term sham-controlled and real-world evidence obtained with radiofrequency renal denervation using the Symplicity Spyral system. This focus reflects the current maturity of the evidence base, as this platform is supported by the most extensive randomized and registry data with follow-up extending to three years. Consequently, the findings should not be interpreted as implying superiority of a specific device. While alternative renal denervation technologies, including ultrasound-based systems such as Paradise (Radiance trials), are under active clinical evaluation, comparable long-term randomized and real-world outcome data are not yet available. Future studies will clarify whether the durability, safety, and clinical impact observed in the present synthesis extend across different technologies and procedural approaches. Importantly, the consistency of blood pressure reduction and safety signals highlighted herein supports the broader concept of renal sympathetic modulation as a durable antihypertensive strategy and an emerging component of cardiovascular risk prevention pathways. Emerging research areas that may further refine patient selection and procedural outcomes include the development of biomarkers of sympathetic overactivity, advanced imaging techniques for mapping renal nerve distribution, and ongoing evaluation of RDN safety in patients with more advanced chronic kidney disease. These represent important directions for future research that may improve the precision of this therapeutic approach.

## CRediT authorship contribution statement

**Dominique Stephan:** Writing – review & editing, Writing – original draft, Validation, Supervision, Project administration, Methodology, Investigation, Formal analysis, Data curation, Conceptualization. **Emma Morisot:** Investigation, Formal analysis, Data curation. **François Bronner:** Visualization, Supervision, Project administration, Investigation, Data curation, Conceptualization. **Eric Prinz:** Visualization, Validation, Supervision, Methodology, Investigation, Formal analysis, Data curation, Conceptualization. **Mihaela Calcaianu:** Validation, Supervision, Investigation, Formal analysis. **Elena-Mihaela Cordeanu:** Writing – review & editing, Writing – original draft, Validation, Supervision, Methodology, Formal analysis, Data curation, Conceptualization.

## Declaration of generative AI and AI-assisted technologies in the manuscript preparation process

During the preparation of this work the authors used Claude (Anthropic) and ChatGPT (OpenAI) in order to assist with literature synthesis, manuscript drafting, language editing, and figure preparation. After using these tools, the authors reviewed and edited the content as needed and take full responsibility for the content of the published article.

## Funding

None.
